# Plk1 Inhibition Causes Post-Mitotic DNA Damage and Senescence in a Range of Human Tumor Cell Lines

**DOI:** 10.1371/journal.pone.0111060

**Published:** 2014-11-03

**Authors:** Denise L. Driscoll, Arijit Chakravarty, Doug Bowman, Vaishali Shinde, Kerri Lasky, Judy Shi, Tricia Vos, Bradley Stringer, Ben Amidon, Natalie D'Amore, Marc L. Hyer

**Affiliations:** Takeda Pharmaceuticals International Co., Cambridge, Massachusetts, United States of America; Cellcuity, United States of America

## Abstract

Plk1 is a checkpoint protein whose role spans all of mitosis and includes DNA repair, and is highly conserved in eukaryotes from yeast to man. Consistent with this wide array of functions for Plk1, the cellular consequences of Plk1 disruption are diverse, spanning delays in mitotic entry, mitotic spindle abnormalities, and transient mitotic arrest leading to mitotic slippage and failures in cytokinesis. In this work, we present the *in vitro* and *in vivo* consequences of Plk1 inhibition in cancer cells using potent, selective small-molecule Plk1 inhibitors and Plk1 genetic knock-down approaches. We demonstrate for the first time that cellular senescence is the predominant outcome of Plk1 inhibition in some cancer cell lines, whereas in other cancer cell lines the dominant outcome appears to be apoptosis, as has been reported in the literature. We also demonstrate strong induction of DNA double-strand breaks in all six lines examined (as assayed by γH2AX), which occurs either during mitotic arrest or mitotic-exit, and may be linked to the downstream induction of senescence. Taken together, our findings expand the view of Plk1 inhibition, demonstrating the occurrence of a non-apoptotic outcome in some settings. Our findings are also consistent with the possibility that mitotic arrest observed as a result of Plk1 inhibition is at least partially due to the presence of unrepaired double-strand breaks in mitosis. These novel findings may lead to alternative strategies for the development of novel therapeutic agents targeting Plk1, in the selection of biomarkers, patient populations, combination partners and dosing regimens.

## Introduction

The disruption of mitotic progression is a commonly used and clinically effective strategy for treating cancer. Nearly six decades after the characterization of the first anti-mitotic agents, the vinca alkaloids (reviewed in [1]), our mechanistic understanding of the effects of these agents on cancer cells continues to evolve.

Initial reports of the mechanism of action of anti-mitotic agents, based on endpoint assays, gave rise to a canonical view that involved a prolonged mitotic arrest followed by apoptosis (reviewed in [2]). Subsequent reports from various researchers were contradictory and often confusing, as some groups reported the canonical mechanism while others reported a transient mitotic delay followed by permanent growth arrest [3] or cell death [4]. In recent years, high-content live-cell imaging techniques have revealed a striking diversity of responses to the same agent across a range of cell lines [5] [6] [7], and importantly, heterogeneous, stochastic responses within the same cell line [5]. These more comprehensive characterizations, with real-time techniques, have in general yielded a more diverse picture of the duration and downstream consequences of mitotic arrest.

In addition to apoptosis, another common outcome of mitotic disruption has been shown to be cellular senescence, an irreversible terminal growth arrest that occurs as a result of cellular stress or DNA damage [8]. Cellular senescence is distinguished by morphological changes, such as enlarged cellular size and increased vacuolization, as well as biochemical changes, such as the induction of senescence-associated β-galactosidase (SA-β-gal) activity [9] [10] [11] and a transient rise in levels of the tumor suppressor proteins p53 and p21 [12] [13]. A wide variety of antimitotic agents have been shown to be both strong (discodermolide, alisertib) and weak (taxol, vincristine) inducers of senescence [13] [14]. Senescence has also been reported to be a downstream consequence of agents that cause DNA double-strand breaks [10]. Indeed the DNA double-strand break response has been demonstrated in some settings to be necessary for the induction of senescence [15] [16].

Plk1 is a checkpoint protein whose role spans all of mitosis and includes DNA repair, and appears to be highly conserved in all eukaryotes. Consistent with this wide array of functions for Plk1, disruption of Plk1 function by small-molecule inhibitors, siRNA or dominant negative (kinase-dead) mutant protein expression has led to a variety of mitotic defects, including: mitotic entry delays [17] [18] [19] [20] [21], defects in centrosome maturation [18] [19] [22] and separation [20] [23] [24] [25] [26], mitotic spindle abnormalities such as monopolar spindles [27] [28], shortened spindles [23] [24] [25], and defects in chromosomal alignment [26] [20] [29] [30]. In turn, these defects in spindle organization result in either prolonged mitotic arrest [27] [20] [28] [31] [32] [33] [34] [35], transient mitotic arrest leading to mitotic slippage [28], or failures in cytokinesis [26] [36]. Consistent with these cell-cycle progression defects, an increase in aneuploid cells [36], micronuclei [27], disorganized spindle poles [19] and defects in chromosomal alignment [37] have been reported as a consequence of Plk1 inhibition, as has an increase in apoptosis [32] [33] [34] although in some cases the decrease in proliferative potential has been linked to the induction of growth arrest [25] [29] [31].

Based on the ever growing understanding of anti-mitotic effects for a variety of agents, and on the link between DNA damage and senescence, we hypothesize that Plk1 inhibition leads to DNA damage as a result of mitotic arrest, followed in some settings by cellular senescence. Recently it was shown that Plk1 inhibition, via genetic knock-down, induces senescence in normal human fibroblast cells, partially supporting our hypothesis [38]. In our current report we further explore this hypothesis, using several novel experimental small-molecule inhibitors of Plk1 and anti-Plk1siRNA to examine the cellular consequences of prolonged or transient mitotic arrest, specifically in cancer cells.

## Materials and Methods

### Cell culture

The culture media was purchased from Invitrogen Corporation (Carlsbad, CA). The fetal bovine serum (FBS) was purchased from Hyclone Laboratories (South Logan, Utah). All cell lines were obtained from American Type Culture Collection (ATCC) (Manassas, VA) and were cultured in a humidified incubator at 37°C with 5% CO_2_. HCT116 cells and HT-29 cells (Human colon cancer cells) were grown in McCoys 5A with 10% FBS. A549 cells, Calu-6 cells (Human lung cancer cells), DLD-1 cells and SW480 cells (Human colon cancer cells) were cultured in F-12K, MEM, RPMI1640 and Leibovitz's L-15 supplemented with 10% FBS, respectively.

### FACS analysis

HCT116 and HT-29 cells (1×10^6^/plate) were seeded on 100 mm dishes and cultured at 37°C overnight. The next day, cells were incubated with either MLN0905 or DMSO for the indicated times. Adherent and floating cells were harvested and fixed in 70% ethanol and stored overnight at -20°C. Cells were centrifuged and cell pellets washed with 0.5% BSA in PBS and then stained with γH2AX antibody (Millipore Corporation, Billerica, MA) per the manufacturer's instructions and incubated overnight at 4°C. Pellets were then washed and stained with the Alexa-488 labeled secondary antibody (Molecular Probes, Life Technologies Corporation, Bedford, MA) diluted 1∶150 and incubated for 30 minutes at room temperature. Pellets were washed and re-suspended in propidium iodide and RNaseA in PBS. Cell-cycle distributions were determined using flow cytometry (FACS Calibur, Becton Dickinson, Franklin Lakes, NJ) and analyzed using Winlist software (Verity Software House, Topsham, ME).

### siRNA experiments

Cells were transfected with 10 nM of GL2 (sense, 5′ CGUACGCGGAAUACUUCGA 3′) or Plk1 (sense, 5′CCGAGUUAUUCAUCGAGAC3′) siRNAs using DharmaFECT 2 (DH2) reagent (Dharmacon) in a BioCoat poly-D-lysine(PDL)-coated 60-mm dishes (BD Biosciences) [39]. A reverse transfection was performed as described in [39]. Plates were harvested at the indicated time points.

### Western blot analysis

Cells were seeded in 100 mm dishes (1×10^6^/dish) and cultured at 37°C overnight. The next day, cells were treated with compounds as indicated. At indicated times both floating and adherent cells were collected and whole cell extracts prepared using radioimmunoprecipitation (RIPA) lysis buffer (150 mM NaCl, 50 mM Tris pH 7.5, 1 mM EDTA, 1% NP-40, 1% Na deoxycholic acid and 0.1% sodium dodecyl sulfate [SDS]). RIPA buffer was supplemented with 1 mM phenylmethylsulfonyl fluoride (PMSF), 1× Boehringer “complete” protease inhibitor cocktail, 50 mM NaF, 25 mM beta-glycerophosphate, 50 mM Na orthovanadate, 5 mM 1,10-phenanthroline monohydrate and 250 units/µL benzonase nuclease HC. The Bradford protein assay (DC Protein Assay Kit, Bio-Rad, Laboratories, Waltham, MA) was used to determine protein concentration and 25 µg of lysate was fractionated on 4% to 12% NuPage Bis-Tris gels. Proteins were transferred to nitrocellulose membranes (0.45 µM pore size) and all primary antibodies were diluted in TBS containing 1% bovine serum albumin (BSA) and 0.1% Tween-20. Membranes were incubated overnight with primary antibody at 4°C. Antibodies used include: pS10 H3 (pHisH3) (1∶500, Cell Signaling Technologies, Danvers, MA), cleaved caspase-3 (1∶1000, Cell Signaling Technologies, Danvers, MA), PARP (1∶1000, Cell Signaling Technologies Danvers, MA), pS139 H2AX (γH2AX) (1∶4000, Millipore Corporation, Billerica, MA), p53 (1∶2000, Santa Cruz Biotechnology, Santa Cruz, CA), p21WAF1 (1∶10,000, Neomarkers Inc., Fremont, CA), actin (1∶20,000, Abcam plc, Cambridge, UK) and Plk1 (1∶1000, Invitrogen Corporation, Carlsbad, CA), γ-tubulin (1∶2000, Abcam plc, Cambridge, UK), p-Chk1(S317) (generated by Millennium Pharmaceuticals, Inc.), p-Chk2(T68) (1∶1000, Epitomics Inc, Burlingame, CA), goat α-rabbit IgG-HRP (1∶2000, Santa Cruz Biotechnology, Santa Cruz, CA), and goat α-mouse IgG-HRP (1∶4000, Santa Cruz Biotechnology, Santa Cruz, CA). ECL reagent (Amersham, GE Healthcare Life Sciences, Pittsburgh, PA) was used to detect proteins of interest.

### Cell culture immunofluorescence (IF)

Cells were grown in 96-well tissue culture plates and treated with 0.1% DMSO (control), MLN0905 or BI 2536 for the indicated times. Cells were fixed in 4% PFA (paraformaldehyde), and incubated at 4°C with primary antibodies, γH2AX (Millipore Corporation, Billerica, MA) and pHisH3 (Cell Signaling Technologies, Danvers, MA) overnight. Cells were washed and incubated with secondary antibodies conjugated to Alexa 594 and Alexa 488 (Molecular Probes, Life Technologies Corporation, Bedford, MA) for 1 hr with Hoechst. Plates were acquired on the Discovery-1 HCS System (Molecular Devices LLC, Sunnyvale, CA) and analyzed with the MetaXpress CellScoring Application Module (Molecular Devices LLC, Sunnyvale, CA).

### β-Galactosidase staining

β-gal staining (both tissue culture and *in vivo*) were performed as previously described [13]. In brief, frozen tumor samples were sectioned at 4–6 µm and stained for β-galactosidase (β-gal) using the β-gal staining kit (Catalog #G1041-76, US Biological, Marblehead, MA) and counterstained with Hematoxylin. Slides were scanned at 20× with an Olympus BX61VS microscope equipped with an automated XY stage. A 1 mm^2^ region of interest was selected and analyzed using MetaMorph software (Molecular Devices LLC, Sunnyvale, CA). Senescence was measured as the percentage of positive β-gal signal versus total cell area.

### In vivo pharmacodynamic studies

Female nude mice (Taconic) bearing subcutaneous HCT116 xenograft tumors were used for the PD studies (n = 3 animals/group). At the indicated time points animals were euthanized using carbon dioxide and tumor tissue collected for PD analysis. Tumors were harvested and divided into two parts, half frozen on dry ice (and stored at −80°C) for β-gal staining and half fresh formalin fixed/paraffin embedded (FFPE) for IHC analysis. All animal studies were approved by the Institutional Animal Care and Use Committee (IACUC) at Takeda Pharmaceuticals Inc.

### Pharmacodynamic analysis

The immunohistochemistry methodology was previously described for pHisH3 [13], [40]. For γH2AX staining tumor samples were harvested and fixed in 10% neutral-buffered formalin. Immunohistostaining was performed using a Ventana Discovery XT Autostainer platform (Ventana Medical Systems, Tucson, AZ) on 5 µm thick sections obtained from formalin fixed paraffin embedded tumor blocks. Tumor sections were deparaffinized, followed by epitope unmasking with Cell Conditioning 1 Solution (Ventana Medical Systems, Tucson, AZ). Sections were incubated with γH2AX (pS139) rabbit monoclonal antibody (2212-1, Epitomics Inc, Burlingame, CA) followed by goat anti-rabbit biotinylated antibody (BA1000, Vector Inc, Burlingame CA) as a secondary. A DAB colorimetric detection system (Ventana Medical Systems, Tucson, AZ) was used to identify biomarker expression. Slides were scanned using an Aperio Scanscope XT (Aperio Technologies, Inc, Vista CA), and percent positive γH2AX area was evaluated using Aperio analysis software.

### In vivo efficacy studies

The HCT116 xenograft tumor model was used as previously described [40]. Tumor bearing animals (n = 10/group) were treated orally with Plk inhibitors or vehicle as indicated. MLN0905 and Compound 12b [41] were formulated as previously described [39]. The statistical analysis used has previously been described [39], [41].

## Results

### Plk1 inhibition leads to mitotic delays and DNA damage

**Figure 1:**
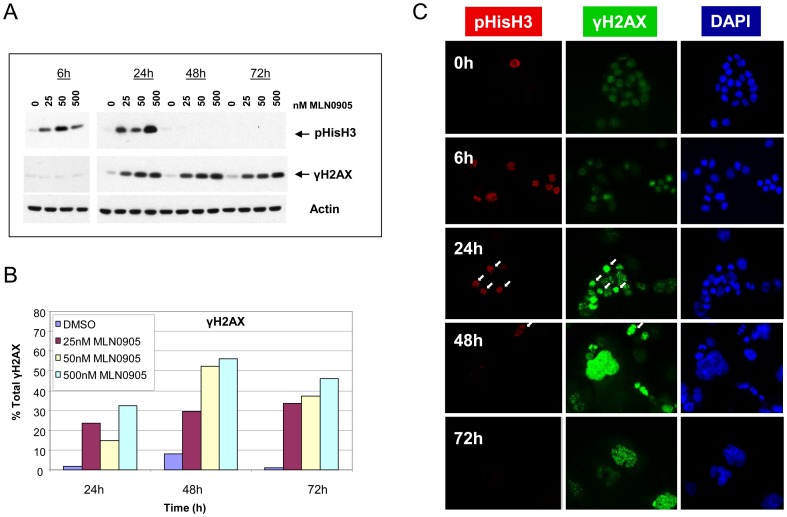
Plk1 inhibition leads to transient mitotic arrest followed by DNA damage in HCT116 cells. Cells were treated with increasing concentrations of MLN0905 and at indicated times analyzed for mitotic arrest (via pHisH3) and DNA damage (via γH2AX). A) Immunoblotting indicates transient mitotic arrest leads to DNA damage (representative blot shown from two independent experiments). B) The same samples used in A) were quantified for DNA damage (γH2AX staining) using FACS analysis. C) In a separate experiment immunofluorescent chemistry was also used to demonstrate mitotic arrest precedes DNA damage (representative images shown from two independent experiments; 50 nM MLN0905 used).

**Figure 2:**
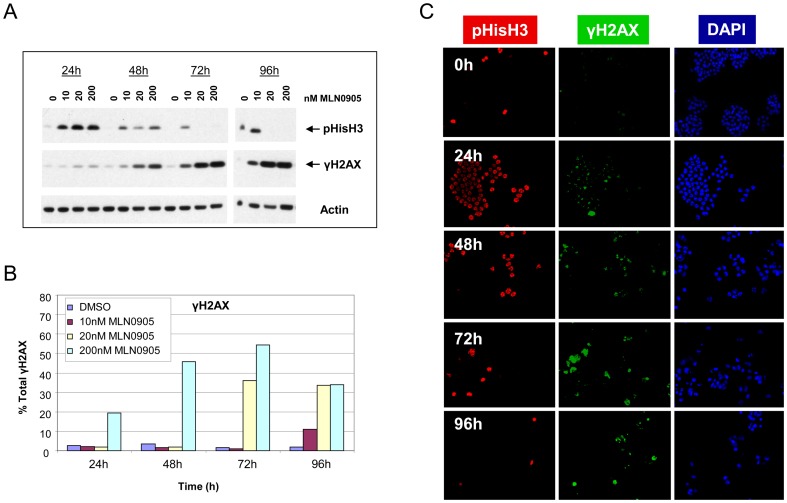
Plk1 inhibition leads to prolonged mitotic arrest followed by detection of DNA damage in HT-29 cells. Cells were treated with increasing concentrations of MLN0905 and mitotic arrest (via pHisH3) and DNA damage (via γH2AX) were analyzed at indicated times. A) Immunoblotting was used to demonstrate prolonged mitotic arrest precedes DNA damage (representative blot shown from two independent experiments). B) The same samples used in A) were quantified for DNA damage (γH2AX) using FACS analysis. C) In a separate experiment immunofluorescent chemistry was also used to demonstrate mitotic arrest (pHisH3) precedes DNA damage (γH2AX) (representative images shown from two independent experiments; 200 nM MLN0905 used).

MLN0905 is a potent experimental small molecule inhibitor of Plk1 [39], [41]. MLN0905 was used to study the induction of mitotic delays and DNA damage following Plk1 inhibition in cells. HCT116 cells (Figure 1) or HT-29 cells (Figure 2) were treated with varying concentrations of MLN0905 over the course of 3 days. At appropriate times cells were harvested for analysis by western blot (WB, Figure 1A, 2A), fluorescent cell sorting (FACS, Figure 1B, 2B) and immunofluorescence (IF, Figure 1C, 2C) to observe effects on cell cycle and DNA damage response markers.

In HCT116 cells, pronounced mitotic arrest occurs out to 24 hours, as judged by the appearance of pS10 H3 (pHisH3), a canonical marker of mitosis (Figure 1A and C). This transient mitotic arrest is also observed by real-time phase-contrast microscopy (Movie S2 - cells were treated with a PLK1 inhibitor, Movie S1 - vehicle control). DNA damage, as measured by p139 γH2AX WB (Figure 1A), FACS (Figure 1B) or IF staining (Figure 1C) occurs at or after 24 hours. In this cell line, the accumulation of DNA damage is associated with mitotic cells (characterized by condensed DNA staining and strong pHisH3 localization; arrows, Figure 1C), and DNA damage foci are also present in non-mitotic cells at later time-points (48–72 hours). DNA damage was confirmed in this line using the DNA damage markers pChk2-T68 and pChk1-S317 (Figure S1).

In HT-29 cells (Figure 2), pronounced mitotic arrest occurs out to 48 hours as observed by pHisH3 (Figure 2A, 2C) and by real-time phase-contrast microscopy (Movie S4 - cells were treated with a PLK1 inhibitor, Movie S3 - vehicle control). DNA damage occurs after 24 hours, increasing with time (Figure 2A, 2C). Again in this cell line, DNA damage is often observed in mitotic cells (Figure 2C), although DNA damage foci are also observed in non-mitotic cells at later time-points (72 and 96 hours). DNA damage was confirmed in the HT-29 cell line using the DNA damage marker pChk2-T68 (Figure S1).

Although the length of mitotic delay in HCT116 and HT-29 cells differs, as indicated by pHisH3, the appearance of mitotic arrest precedes that of DNA damage (γH2AX) in both cell lines. For example in the HCT116 cells, the order of events is evident at 6 hours where there is an increase in pHisH3, yet no detectable γH2AX. Only after 24 h is there any appearance of γH2AX that persists through the remainder of the time course. It is interesting to note that in HCT116 or HT-29, the levels of γH2AX are constant or increasing, respectively, at the latter time points regardless of the length of the mitotic delay.

Although MLN0905 is a highly-specific Plk1 inhibitor, we confirmed the above observations were Plk1 specific using two additional Plk1 inhibitor approaches. The first method utilized an additional small molecule Plk1 inhibitor from a different chemical scaffold, BI 2536 [42], and the second method used a genetic knock-down strategy targeting Plk1 with RNAi. First, HCT116 cells were treated with the BI 2536 compound then assayed for mitotic arrest and DNA damage using western blotting and immunofluorescence. Consistent with our MLN0905 findings, the BI 2536 compound induced mitotic arrest followed by DNA damage (Figures S2).

**Figure 3:**
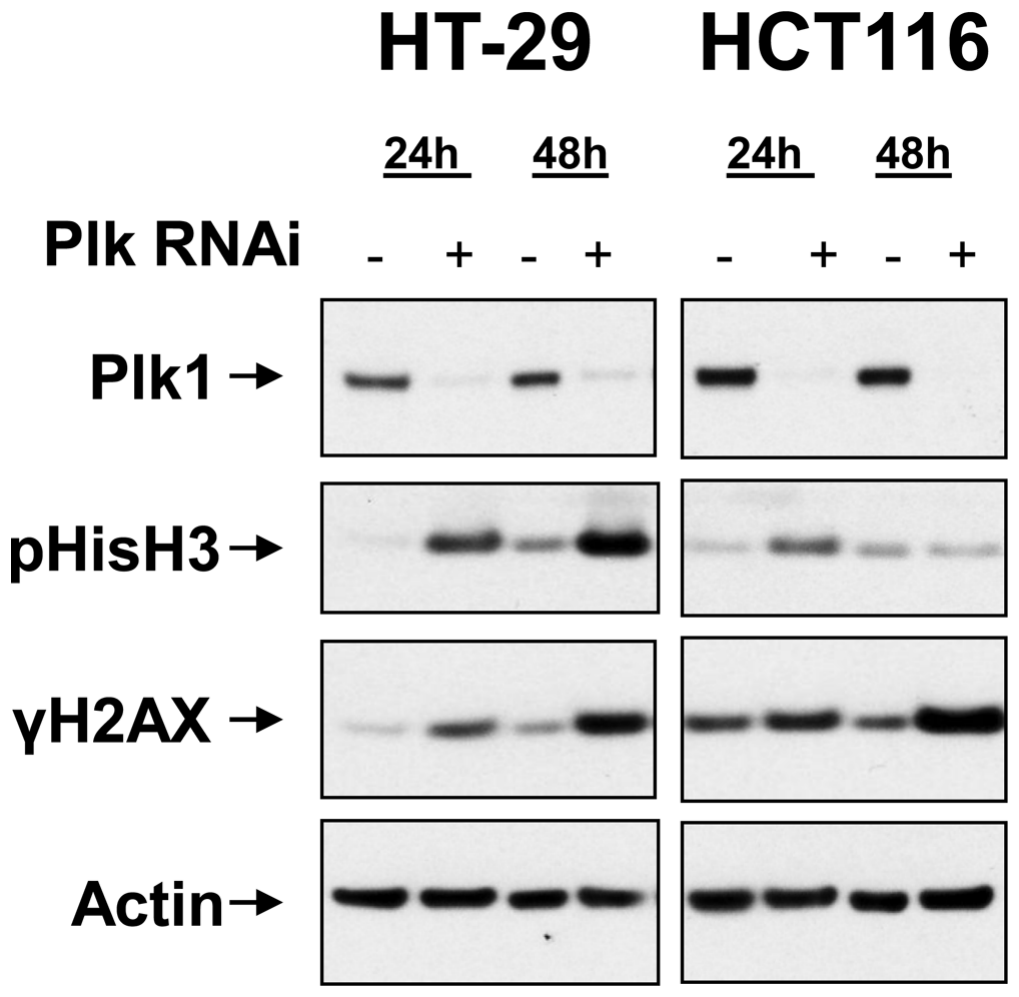
RNAi (10 nM final concentration) directed against Plk1 leads to mitotic arrest followed by increased DNA damage. In HT-29 cells, a prolonged mitotic arrest (pH3) is followed by an increase in DNA damage (γH2AX). In HCT116 cells, transient mitotic arrest (pHisH3) is followed by an increase in DNA damage (γH2AX).

To confirm the observed DNA damage was Plk1-mediated, RNAi (10 nM final concentration) directed against Plk1 was used in both cell lines to investigate the effects of Plk1 loss-of function. Consistent with the findings using small molecule inhibition of Plk1, in HT-29 cells (Figure 3, left panels), a prolonged mitotic arrest (pHisH3) is followed by an increase in DNA damage (γH2AX), whereas in HCT116 cells (right panels), a transient mitotic delay (pHisH3) is followed by an increase in DNA damage (γH2AX).

**Figure 4:**
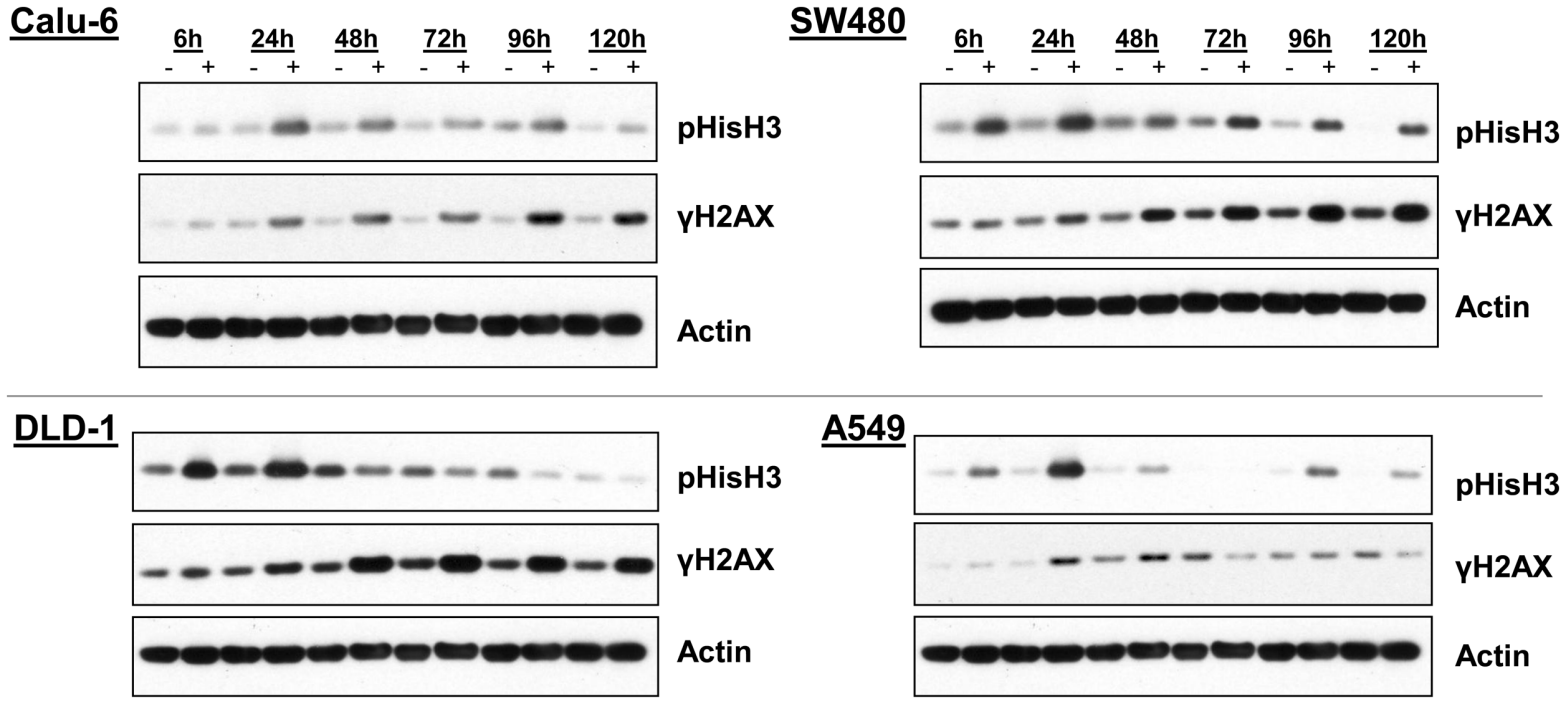
Plk1 inhibition leads to mitotic arrest and DNA damage in a wide range of human cancer cell lines. Cells were treated with MLN0905 (Calu-6, 10 nM; DLD-1, 90 nM; A549, 30 nM; SW480, 20 nM) for the indicated times and immunoblotting was used to monitor mitotic arrest (pHisH3) and DNA damage (γH2AX).

Next, additional cell lines were evaluated for effects on mitotic arrest and DNA damage following treatment with MLN0905 (Figure 4). In DLD-1, Calu-6, SW480 and A549 cells, Plk1 inhibition using MLN0905 once again led to a mitotic arrest that lasted out to varying lengths of time in each cell line, ranging from 24 to 120 hours, as measured by pHisH3 western blotting. In each cell line, DNA damage (as measured by γH2AX) was once again observed following the initial induction of mitotic arrest (concomitant in the Calu-6 cells). In all cell lines tested, DNA damage levels were highest at time points following the peak of induction of mitotic arrest, once again consistent with a model where DNA damage induction occurred either in mitosis or post-mitotically.

### Plk1 inhibition, in vitro, leads to cellular senescence

**Figure 5:**
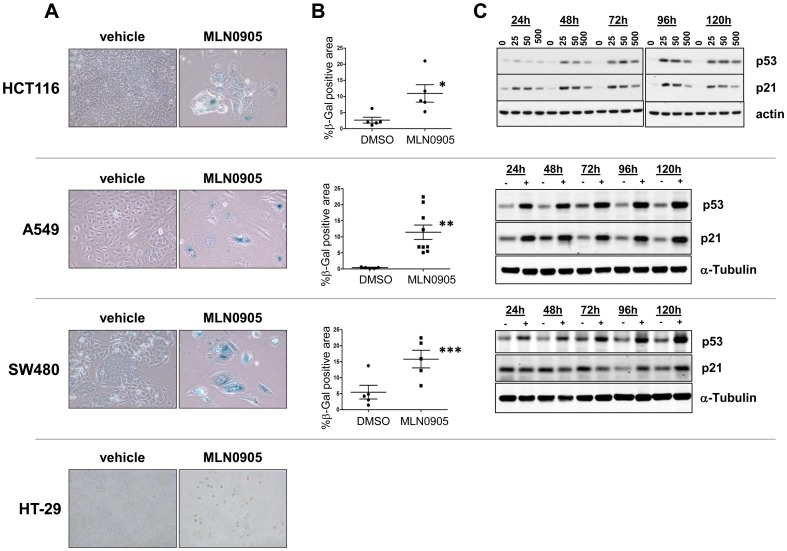
Plk1 inhibition leads to senescence in multiple cell lines. A) Cells were treated for two weeks with MLN0905 (50 nM for HCT116, 30 nM for A549, 20 nM for SW480 and HT-29) and then stained for β-galactosidase activity. B) β-galactosidase staining was then quantified in senescent cells and represented as % area (shown is mean ±SD; *p = 0.0159, **0.0033 and ***0.032 by a two-tailed Mann-Whitney test).

As cellular senescence has been reported in the literature as a commonly occurring molecular consequence of DNA damage [15], [16] we investigated the effects of Plk1 inhibition on the induction of senescence in a subset of the cell lines. HCT116, A549, SW480 and HT-29 cells were chronically treated with MLN0905 for two weeks continuously with one change of medium. Cells were then stained with β-galactosidase for 24 hours. Strong β-galactosidase staining was observed in HCT116, A549 and SW480 cells, accompanied in each case by two other morphological readouts of senescence, a dramatic increase in cellular area and a flattened cellular morphology (Figure 5A) (13). The β-galactosidase positive areas were quantified using an image analysis algorithm as described in the materials and methods and found to be significantly different from vehicle controls (Figure 5B). In contrast, the p53-deficient HT-29 cells did not display β-galactosidase staining or a flattened senescent-like morphology.

A secondary Plk1 inhibitor was used to confirm presence of senescence in the HCT116 cells. Cells were continuously treated with the BI 2536 compound for 21 days then assayed for senescence using cellular morphology and β-galactosidase staining readouts. Consistent with the results using MLN0905, HCT116 cells displayed a large-flat cellular morphology and stained positive for β-galactosidase activity (Figure S2C).

The senescent phenotype in the HCT116, A549 and SW480 cells was then further analyzed using the biochemical markers p53 and p21, as both proteins have been previously demonstrated by us (13) and others [10] to be strongly up-regulated upon induction of senescence. Upon treatment with MLN0905, HCT116 and A549 cells showed a strong induction of both p53 and p21 out to 120 hours (Figure 5C). In SW480 cells, p53 induction was apparent at all time points while p21 induction was observed at the 96 and 120 hour time points. In contrast, for those cell lines where senescence was not seen, the up-regulation of p53 and p21 was not observed. DLD-1 cells are p53 mutant and p21 levels in this cell line remain unchanged with MLN905 treatment (data not shown) while HT-29 and Calu-6 cells are p53 and p21 null.

Although Plk1 inhibition via MLN0905, leads to senescence in a range of cell lines, we still note that apoptosis is also a terminal outcome observed *in vitro* (Figure S3). Six different cell lines (HT-29, DLD-1, Calu-6, HCT116, SW480 and A549) were treated with MLN0905 out to 120 hours and assayed for their apoptotic response using cleaved PARP and cleaved caspase-3. Plk1 inhibition led to a strong apoptotic response in some cell lines (HT-29, DLD-1, and Calu-6) while inducing weaker-to-no detectable apoptotic responses in other cell lines (HCT116, SW480, A549). It is interesting to note those cell lines where senescence was demonstrated by us as a terminal outcome of Plk1 inhibition were the ones with weak apoptotic responses. To further validate the above observations RNAi directed against Plk1 induced PARP cleavage in HT-29 cells but not in HCT116 cells (data not shown).

### Plk1 inhibition in vivo leads to mitotic arrest, DNA damage, senescence, and tumor stasis

**Figure 6:**
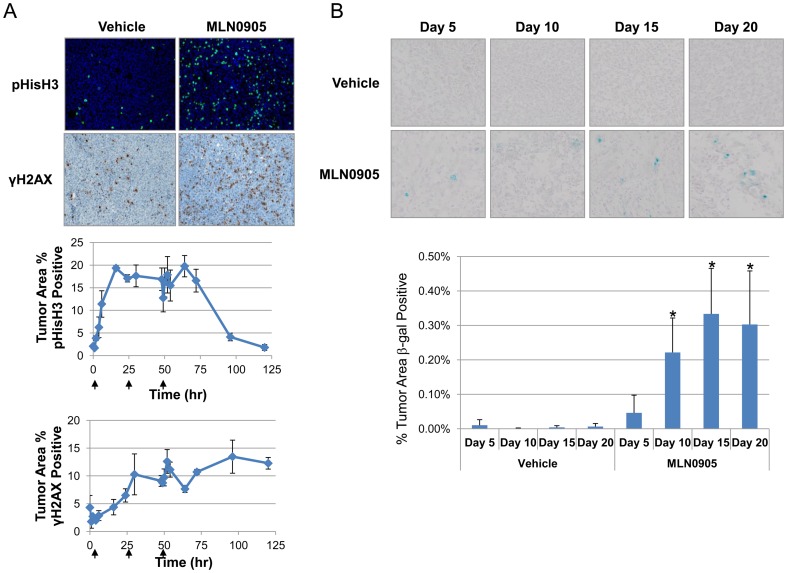
*In vivo*, Plk1 inhibition leads to mitotic arrest, DNA damage and senescence. A) HCT116 tumor bearing mice were dosed orally once a day with 23.3 mg/kg of MLN0905. Tumor tissues were harvested and stained for pHisH3 (green staining) and γH2AX (brown staining). Representative tumor sections are shown with either vehicle or MLN0905 treatment 24 hours after the first dose. pHisH3 and γH2AX signals were quantified (see graph, shown is mean ±SD) and arrows indicate when doses were delivered. B) Tumor tissues were harvested at the indicated times and stained for β-galactosidase activity. Images shown indicate β-galactosidase staining in the MLN0905 treated tumors but not in the time-matched vehicle controls. Staining was quantified and is represented as % tumor area positive (see graph, shown is mean ±SD; *p≤0.04, two-tailed paired t-test).

Having demonstrated Plk1 inhibition leads to mitotic delay, DNA damage, apoptosis and senescence in cultured cells, we next evaluated the outcomes of Plk1 inhibition *in vivo*. Nude mice bearing HCT116 xenograft tumors were used to study the effects of Plk1 inhibition following both acute and chronic treatment of MLN0905. In the first experiment, mice bearing HCT116 tumors were dosed acutely, i.e. 3 consecutive days (QDx3), with MLN0905 (23.3 mg/kg) and tumor tissues were analyzed for mitotic arrest (pHisH3) and DNA damage (γH2AX). This analysis revealed robust pHisH3 and γH2AX staining in MLN0905 treated tumors with the signal lasting for several days (Figure 6A). Next, HCT116 tumor bearing mice were treated chronically with MLN0905 (23.3 mg/kg) for 3 weeks on a QDx3/week schedule and tumor tissues were assayed for senescence at various time points throughout the duration of the study. Using β-galactosidase as a senescence readout, MLN0905 treated tumors stained positive for senescence compared to time-matched vehicle control tumors (p = 0.04, two-tailed Mann-Whitney U-test, Figure 6B). Maximum β-galactosidase staining was detected between days 10 and 20. The timing of detectable senescence in vivo is consistent with what others have found using similar anti-mitotic agents which also induce senescence [13].

An additional small molecule Plk1 inhibitor, Compound 12b - structurally similar but not identical to MLN0905, was also used to evaluate the pharmacodynamic effects of Plk1 inhibition *in vivo*. Compound 12b (previously described in [41]) phenocopies the effect of MLN0905 treatment *in vitro* in HCT116, with the induction of mitotic delay, followed by DNA damage and up-regulation of p53 and p21 (Figure S4A). As with MLN0905, treatment with Compound 12b *in vitro* resulted in detectable β-gal staining (Figure S4B and S4C). Compound 12b was then dosed orally *in vivo* into mice bearing HCT116 xenograft tumors on a Q2Dx2/week schedule (Monday, Wednesday) for 3 consecutive weeks using 50 mg/kg. On day 21, i.e. 4 days following the last dose, tumor tissues were harvested and analyzed for mitotic arrest (pHisH3) and DNA damage (γH2AX). No increase in pHisH3 was detected (data not shown) suggesting tumor cells were not cycling. In contrast, compound 12b treatment resulted in a significant increase in the level of DNA damage observed via γH2AX immunohistochemistry (Figure S5). This is particularly striking considering these animals received their last dose of Compound 12b four days prior to the DNA damage analysis.

As a further biological readout of senescence, we examined the effect of prolonged treatment with Plk1 inhibitors on tumor morphology *in vivo*. Tumor tissues (six for the control and three for the treated, each sample taken from a different animal in the dose group) were stained with H&E on day 21 and assayed for cell size and morphology. Compound 12b treatment resulted in a senescent phenotype, i.e. increased cellular and nuclear size (Figure S6).Five randomly selected images from each tumor tissue were scored for their degree of senescence as manifested by H&E morphology, using a blind scoring approach outlined in Figure S7. Based on this blinded scoring, tumors treated with Compound 12b demonstrated a moderate, but statistically significant, increase in the number of microscopic fields appearing senescent (Figure S6), from 39% in the control (12 out of 30 fields examined in the control) to 86% in the treated sample (13 out of 15 fields examined in the treated sample, p = 0.0038, two-tailed Fisher's Exact Test).

As described above, both Plk1 inhibitors, MLN0905 and Compound 12b, yield senescence as a terminal outcome in the HCT116 xenograft model. Apoptosis has also been observed as a Plk1 terminal outcome in the HCT116 model [43]. We evaluated the anti-tumor effect of both MLN0905 and Compound 12b in the HCT116 xenograft model. HCT116 tumor bearing mice were dosed for 3 weeks with MLN0905 and Compound 12b. Strong anti-tumor activity was observed using both small molecule Plk1 inhibitors characterized by tumor stasis over the 21 day study (Figure S8).

## Discussion

Several small molecule Plk1 inhibitors have been reported to cause mitotic arrest followed by apoptosis [27] [27], [43]. MLN0905, a potent and selective Plk1 inhibitor, also recapitulates that phenotype. However under closer scrutiny, we find that longer term inhibition of Plk1 leads to varying lengths of mitotic delay, DNA damage and a mixture of cell fates that includes cellular senescence.

This study is the first to report the induction of senescence in response to Plk1 inhibition both *in vitro* and *in vivo*, particularly in cancer cells. This observation is consistent with recent evidence demonstrating induction of senescence for other anti-mitotic agents [12] [13] [11], [12], [13], [14]. Cellular senescence has been demonstrated to be a significant contributor to treatment outcome for other anti-mitotic agents in a preclinical setting [44] [44], [45]. The induction of senescence in tumor cells has further been demonstrated to result in immune-mediated tumor cell clearance (reviewed in [46]).

We further report the strong induction of DNA damage in response to Plk1 inhibition, across a broad range of cell lines. These findings are consistent with the emerging literature for anti-mitotics in general. Prolonged mitotic arrest causes DNA damage [47] [48] possibly due to mechanical stresses generated by the mitotic spindle [49]. DNA damage is also caused post-mitotically [50] [51] as chromosomes are caught in the cleavage furrow formed during mitotic exit. Conversely, unrepaired DNA damage has been shown to cause mitotic arrest [52], and many molecular components of the DNA damage checkpoint and the mitotic arrest checkpoint are shared [31] [53] [31], [53], [54].

Our findings substantially broaden the scope of DNA damage following Plk1 inhibition, which has been previously reported in a narrow context. One previous report studied clones generated from non-transformed diploid cell lines (hTERT-RPE1 and MCF10A) depleted of Plk1, and found that, while eleven clones showed no obvious phenotype, one clone demonstrated DNA damage checkpoint activation [55]. Another report studied a single cell line, HeLa cells, synchronized with a number of blocking agents including double thymidine and hydroxyurea, showing that DNA damage occurred in early S phase in a p53-dependent manner [56]. This finding was corroborated in a different study using Xenopus laevis egg extracts [57]. The use of blocking agents is a limitation of these findings, as cell cycle blocks (including the double thymidine/nocodazole block) have been demonstrated to be directly responsible for DNA double-strand breaks (see, for example, [58]).

In our study all six cell lines (with widely varying genotype and lineage) showed the induction of double-strand breaks, as measured by γH2AX. The downstream activation of the DNA damage checkpoint machinery (pChk1/pChk2) was further confirmed in two lines (HT-29 and HCT116 cells). In nearly every case, the peak of the γH2AX signal occurred subsequent to the peak of the pHisH3 signal, and prior to caspase activation. In three of the cell lines with weak apoptosis, strong DNA damage was still observed. Taken together, these findings support a model where DNA damage occurs as a result of prolonged mitotic arrest, independent of the apoptotic machinery. In terms of genetic backgrounds, we found no association between DNA damage and p53 status. Our finding of DNA damage induction in cell lines with weak apoptotic responses argues against the involvement of the apoptotic cascade in the induction of this damage. Given the extensive evidence supporting a role for Plk1 in G2 in the reactivation of the cell-cycle following DNA damage-activated checkpoint arrest [26] [53] [31], if the damage in our cell lines had occurred at the G1-S boundary, the cells would likely have arrested at G2 due to loss of Plk1 function, never entering mitosis in the first place [31]. The observed outcome would then likely have been DNA damage, but not mitotic arrest. Taken together, our findings support a non-apoptotic and mitotic or post-mitotic contribution to the induction of DNA damage upon Plk1 inhibition. It is worth noting that our work lacks the temporal resolution required to ascertain whether Plk1 inhibition-induced DNA damage is generated as a result of mitotic arrest or mitotic exit. This may form the basis of further investigation by others, and could conceivably vary by cell type.

p53 status (wild type vs mutant) has been shown to regulate cellular fate, i.e. apoptosis vs senescence, following DNA damage [10] [10], [59]. Cell lines with a wild type p53 typically undergo a senescence phenotype whereas cell lines harboring mutant p53 typically undergo apoptosis [10] [59]. Our findings were consistent with these previous reports [10] [59] in that HT-29, DLD-1, and Calu-6 (all p53 mutant/null) underwent apoptosis following DNA damage whereas the HCT116 and A549 cell lines (p53 wt) underwent senescence. The SW480 cell line, although p53 mutant (R273H and P309S), responded to DNA damage by undergoing senescence, however this is likely because mutant p53 in this cell line has been shown to have some wild type functions [60].

In summary, our findings add further nuance to the emerging view of the cellular consequences of Plk1 inhibition. The DNA damage and senescence that occur both as a result of transient and prolonged mitotic delays suggest that the initial model of Plk1 leading to prolonged mitotic arrest followed by apoptosis may represent a special case, derived by chance based on the first cell lines used to characterize Plk1 inhibitors.

As a caveat to our work, it is worth noting that we are unable to provide a quantitative assessment of senescence versus apoptosis for Plk1 inhibition. In order to fully understand the contribution of senescence to Plk1 inhibition, it would require a careful survey of a large number of cell lines, and a quantification of apoptosis versus senescence. Complicating this assessment is the fact that apoptotic cells exist transiently, while senescent cells are persistent. With this in mind, one may require mathematical modeling or other quantitative techniques to make a full assessment of the phenomenon. The current manuscript sought to advance our understanding of the generality of senescence as a mechanism for Plk1 inhibition, but by no means reflects the final statement on the matter, and it would be an interesting follow-up publication to flesh out the relative quantitative contributions of apoptosis versus senescence.

Our work leads to a more complete picture of the cellular consequences of Plk1 inhibition and has several practical implications in the development of Plk1 inhibitors. To the extent that DNA damage resulting from Plk1 inhibition contributes to the resultant loss of viability, it can be leveraged in the rational development of small molecule inhibitors. Plk1 inhibition-induced DNA damage can be exploited for indication selection, appropriate combination agents, and in clinical biomarkers focusing on this damage phenotype. Being aware of the induction of senescence (a clinically relevant terminal outcome) in response to Plk1 inhibition helps prevent apoptotic biomarkers from being over-interpreted in the clinic while also helping focus attention on stable disease as a mechanistically relevant outcome of Plk1 inhibition.

## Supporting Information

Figure S1 **Plk1 inhibition leads to mitotic arrest and DNA damage in HCT116 and HT-29 cells.** Cells were treated with increasing concentrations (nM) of MLN0905 and immunoblotting was used to evaluate mitotic arrest (pHisH3) and DNA damage (γH2AX, pChk2-T68, pChk1-S317).
(TIF)

Figure S2 **The small molecule Plk1 inhibitor BI 2536 was found to induce mitotic arrest, DNA damage and senescence in HCT116 cells.** A) Western blotting was used to demonstrate an increase in pHisH3 (mitotic arrest) following BI 2536 compound treatment. DNA damage, as measured by γH2AX, followed mitotic arrest in this cell line. B) DNA damage (γH2AX) was quantified in the HCT116 cells following BI 2536 treatment and immunofluorescent staining. At 48 hours, a>35-fold increase in DNA damage was observed. C) HCT116 cells were continuously treated with 400 nM BI 2536 compound for 21 days changing the medium every three days. Cells were then stained for β-galactosidase activity. A representative field of view is shown at 20× magnification.
(TIF)

Figure S3 **Plk1 inhibition leads to a strong apoptotic response in select cell lines (HT-29, DLD-1, and Calu-6) while inducing weak-to-no apoptotic responses in other cell lines (HCT116, SW480, A549).** Cells were treated with MNL0905 (HT-29, 20 nM; DLD-1, 90 nM; Calu-6, 10 nM; HCT116, 50 nM; SW480, 20 nM; and A549, 30 nM) and immunoblotting was used to analyze cleaved caspase-3 (gray triangles) and PARP (black triangles) at the indicated times. Actin was used as a loading control.
(TIF)

Figure S4 **The Plk1 inhibitor Compound 12b induces mitotic arrest, DNA damage, and senescence in cultured HCT116 cells.** A) Cells were treated with 50 nM Compound 12b and immunoblotting was used to assay for mitotic arrest (pHisH3), DNA damage (γH2AX) and senescence biomarkers (p53 and p21). Alpha-tubulin was used as a loading control. Results indicate Compound 12b phenocopies MLN0905. B) Cells were treated for two weeks with 50 nM Compound 12b and assayed for senescence using β-galactosidase and cellular morphology. Compound 12b treatment resulted in β-galactosidase staining and flattened, large cellular morphology. C) β-galactosidase staining was then quantified in senescent cell lines and represented as % area (shown is mean ±SD; p = 0.0317, two-tailed Mann-Whitney U-test).
(TIF)

Figure S5 **HCT116 tumor bearing mice were orally treated with Compound 12b (or vehicle) for 3 weeks using 50 mg/kg on a Q2Dx2/week schedule in A).** Four days after the last dose (day 21) tumors were harvested and stained for γH2AX (brown staining). B) Data in the graph indicates a statistically significant (shown is mean ±SD; p = 0.0286, two-tailed Mann-Whitney U-test) increase in γH2AX staining following Compound 12b treatment.
(TIF)

Figure S6 **In a separate experiment HCT116 tumor bearing animals were treated orally with 50 mg/kg of Compound 12b on a Q2Dx2/week schedule for 3 weeks.** Tumor tissues were harvested at the end of study (day 21) and stained with H&E to assay cell size. Compound 12b treated cells were substantially larger compared to time matched controls. Using a scoring system (see Figure S7), cell size was quantified on a scale from 0–2 with 2 being the largest and 0 being the smallest. Quantification of the data indicate the Compound 12b-treated tumor cells were larger (p = 0.0038, two-tailed Fisher's Exact Test). Values in the graph represent total tumor cell counts from 5 random fields for each tumor, 3 tumors/treatment).
(TIF)

Figure S7 **H&E sections showing guidelines for blind scoring of senescence morphology.** Four different images were scored in a blinded manner, appearing in random order. Each image was graded on a scale of 0 to 2, with 0 being clearly non-senescent, 1 showing some signs of senescent morphology, and 2 showing clear signs of senescent morphology. The morphological criteria used to establish the scale were: cell size, nuclear size and the degree of vacuolization present in each image.
(TIF)

Figure S8 **In an efficacy study HCT-116 tumor bearing mice were treated separately with A) MLN0905 and B) Compound 12b using 23.3 mg/kg (QDx3/week) and 50 mg/kg (Q2Dx2/week) for 3 weeks, respectively.** Tumor size was measured using vernier calipers and anti-tumor activity was assayed on day 21 of the study. Both MLN0905 and Compound 12b induced significant anti-tumor activity compared to vehicle control (p<0.001). Data shown indicate average tumor volume ±SEM.
(TIF)

Movie S1 **Untreated HCT116 cells were used as a control and time lapsed video microscopy was used to visualize cells out to 5 days.**
(AVI)

Movie S2 **HCT116 cells were treated with the PLK1 small molecule inhibitor BI 2536 (10 nM) and time lapsed video microscopy was used to visualize cells out to 5 days.** The predominant outcome is a transient mitotic arrest, followed by an exit from mitosis (presumably through a mitotic slippage mechanism), followed in many cases by senescence.
(AVI)

Movie S3 **Untreated HT-29 cells were used as a control and time lapsed video microscopy was used to visualize cells out to 72 hours.**
(AVI)

Movie S4 **HT-29 cells were treated with the PLK1 small molecule inhibitor BI 2536 (10 nM) and time lapsed video microscopy was used to visualize cells out to 72 hours.** The primary observation is prolonged and durable mitotic arrest followed by abrupt apoptosis.
(AVI)
