# Supplementary figures and images for: Plk1 Inhibition Causes Post-Mitotic DNA Damage and Senescence in a Range of Human Tumor Cell Lines
Source: PLoS One. 2014 Nov 3;9(11):e111060. doi: 10.1371/journal.pone.0111060 (PMC4218841; doi:10.1371/journal.pone.0111060)

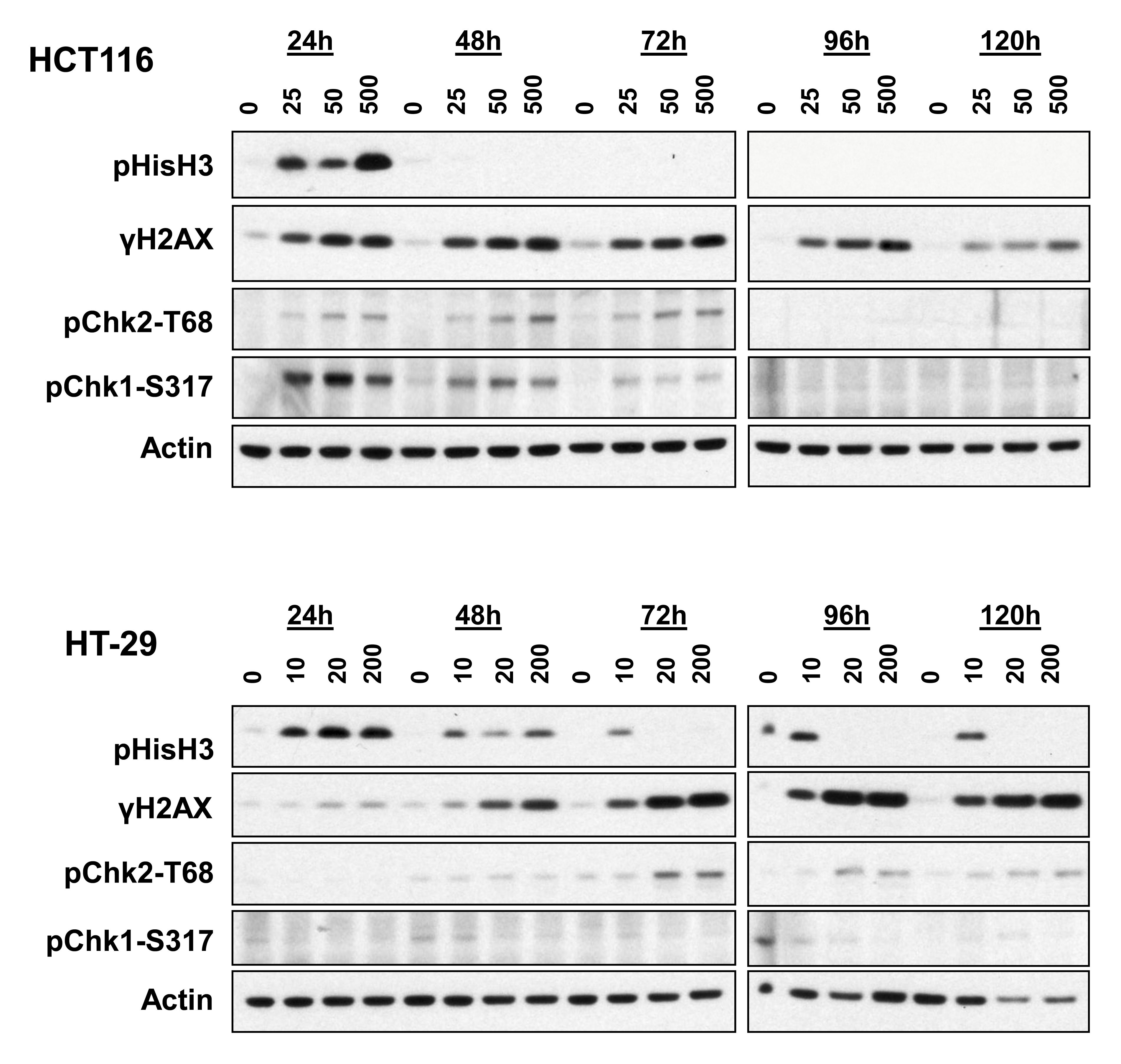

Supplement: Figure S1 — Plk1 inhibition leads to mitotic arrest and DNA damage in HCT116 and HT-29 cells. Cells were treated with increasing concentrations (nM) of MLN0905 and immunoblotting was used to evaluate mitotic arrest (pHisH3) and DNA damage (γH2AX, pChk2-T68, pChk1-S317). (TIF) [file pone.0111060.s001.tif]

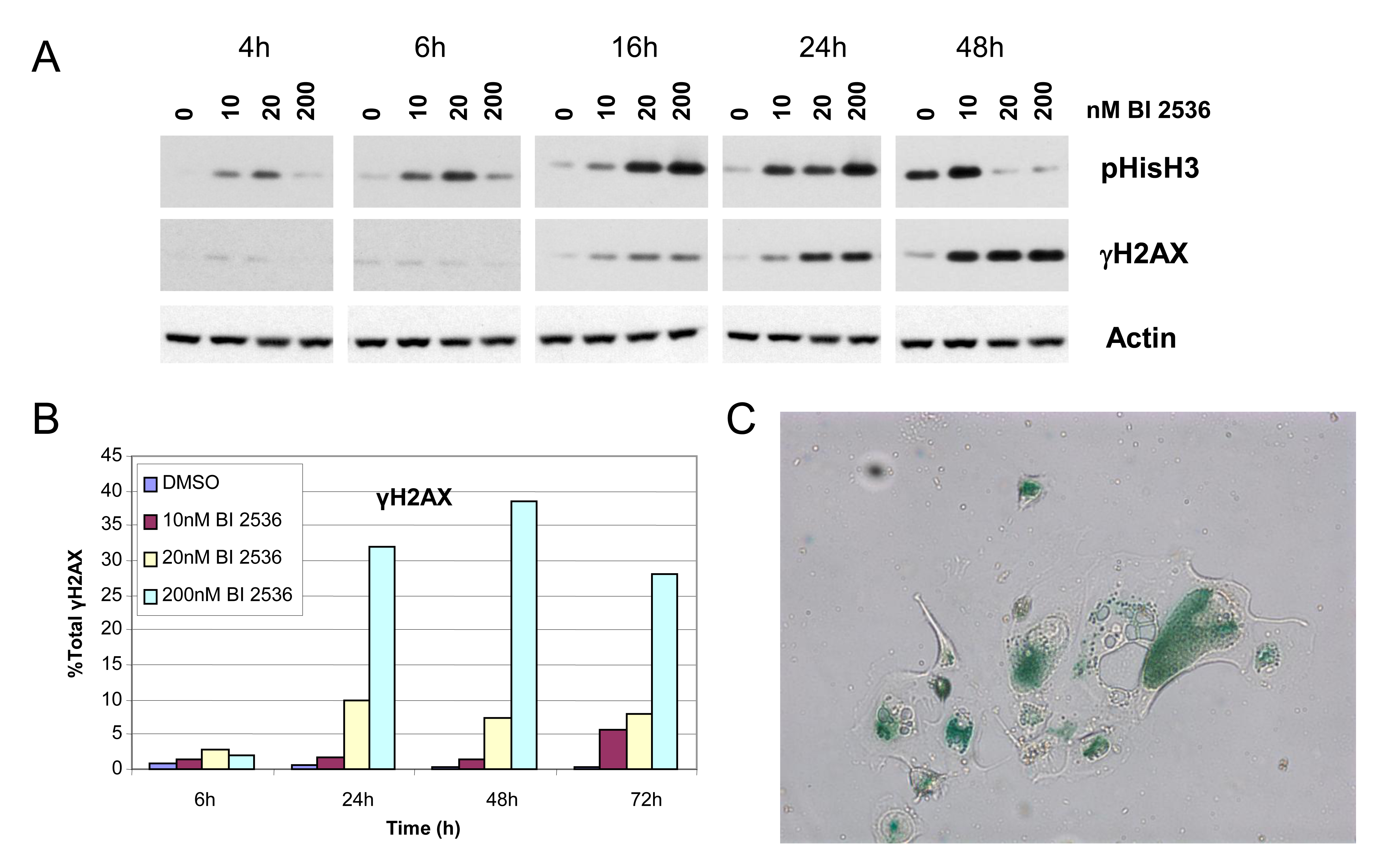

Supplement: Figure S2 — The small molecule Plk1 inhibitor BI 2536 was found to induce mitotic arrest, DNA damage and senescence in HCT116 cells. A) Western blotting was used to demonstrate an increase in pHisH3 (mitotic arrest) following BI 2536 compound treatment. DNA damage, as measured by γH2AX, followed mitotic arrest in this cell line. B) DNA damage (γH2AX) was quantified in the HCT116 cells following BI 2536 treatment and immunofluorescent staining. At 48 hours, a>35-fold increase in DNA damage was observed. C) HCT116 cells were continuously treated with 400 nM BI 2536 compound for 21 days changing the medium every three days. Cells were then stained for β-galactosidase activity. A representative field of view is shown at 20× magnification. (TIF) [file pone.0111060.s002.tif]

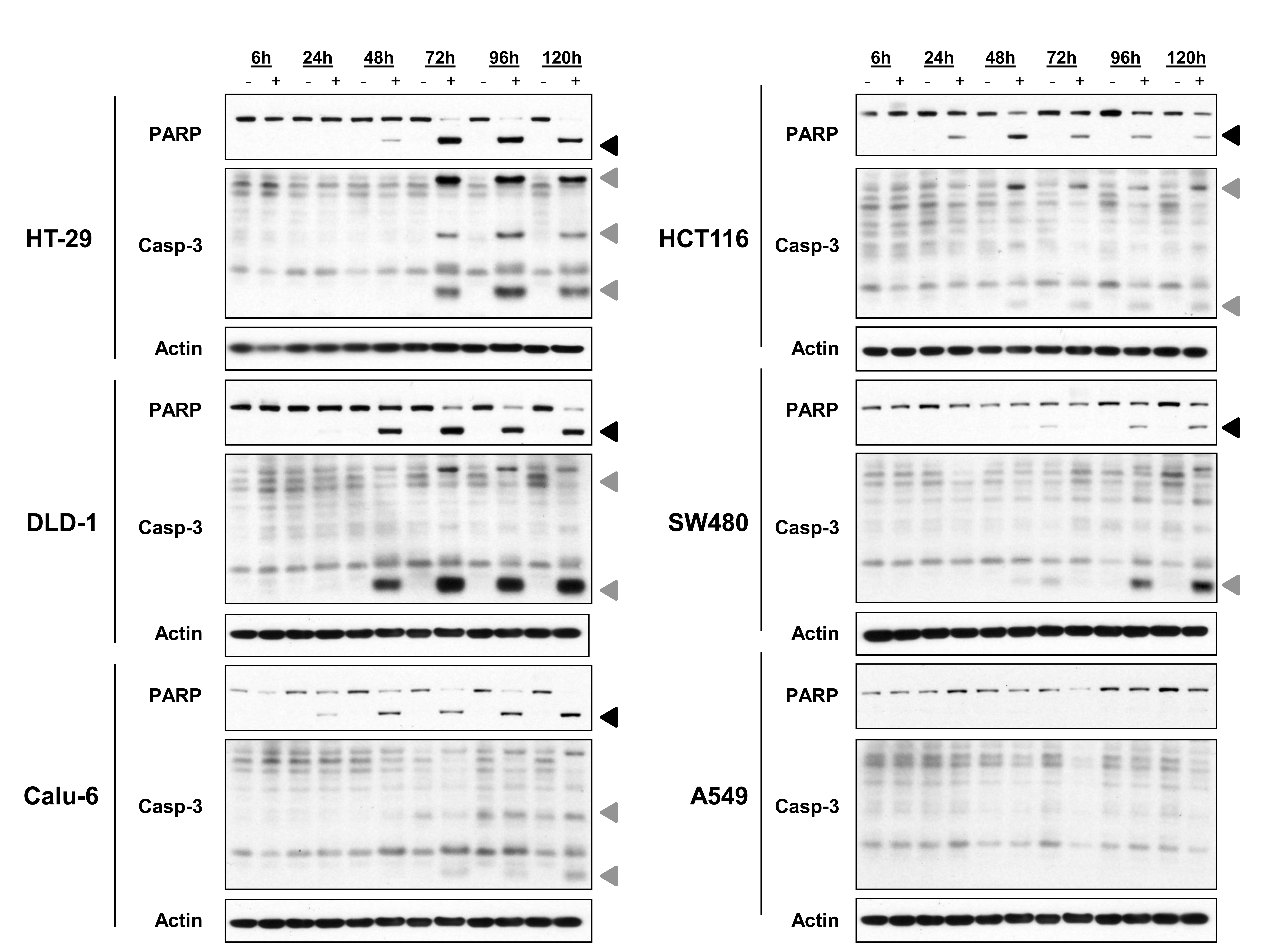

Supplement: Figure S3 — Plk1 inhibition leads to a strong apoptotic response in select cell lines (HT-29, DLD-1, and Calu-6) while inducing weak-to-no apoptotic responses in other cell lines (HCT116, SW480, A549). Cells were treated with MNL0905 (HT-29, 20 nM; DLD-1, 90 nM; Calu-6, 10 nM; HCT116, 50 nM; SW480, 20 nM; and A549, 30 nM) and immunoblotting was used to analyze cleaved caspase-3 (gray triangles) and PARP (black triangles) at the indicated times. Actin was used as a loading control. (TIF) [file pone.0111060.s003.tif]

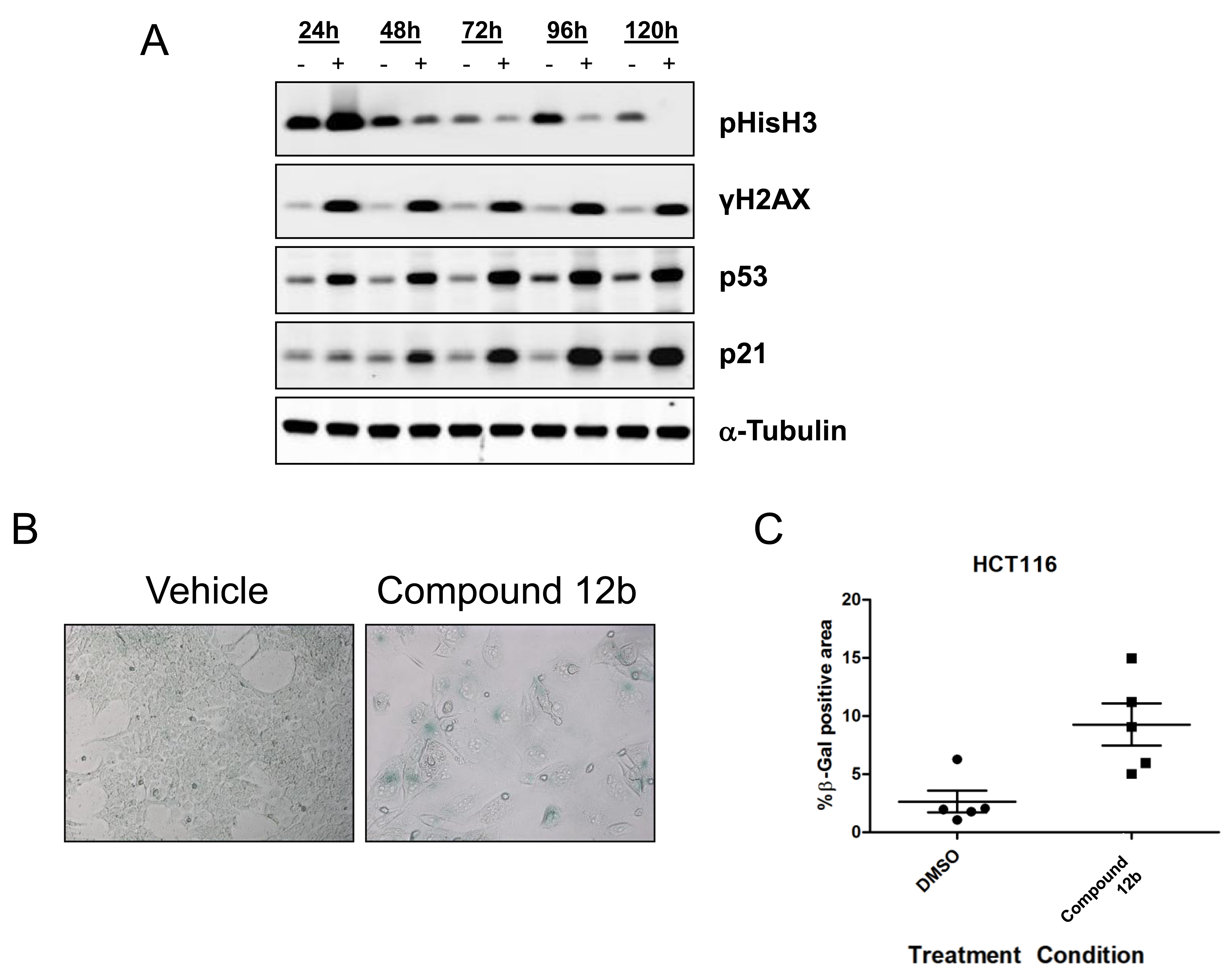

Supplement: Figure S4 — The Plk1 inhibitor Compound 12b induces mitotic arrest, DNA damage, and senescence in cultured HCT116 cells. A) Cells were treated with 50 nM Compound 12b and immunoblotting was used to assay for mitotic arrest (pHisH3), DNA damage (γH2AX) and senescence biomarkers (p53 and p21). Alpha-tubulin was used as a loading control. Results indicate Compound 12b phenocopies MLN0905. B) Cells were treated for two weeks with 50 nM Compound 12b and assayed for senescence using β-galactosidase and cellular morphology. Compound 12b treatment resulted in β-galactosidase staining and flattened, large cellular morphology. C) β-galactosidase staining was then quantified in senescent cell lines and represented as % area (shown is mean ±SD; p = 0.0317, two-tailed Mann-Whitney U-test). (TIF) [file pone.0111060.s004.tif]

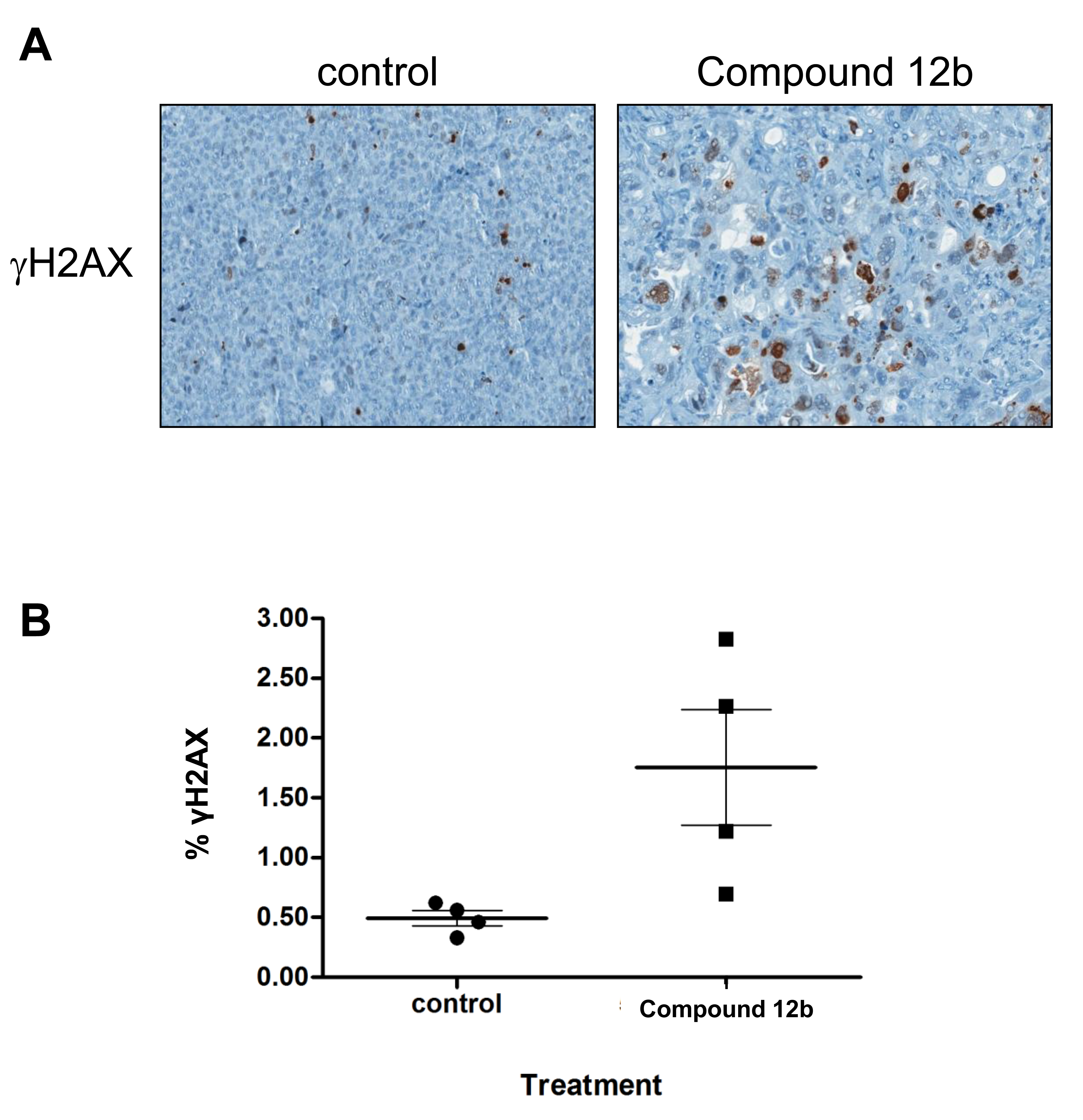

Supplement: Figure S5 — HCT116 tumor bearing mice were orally treated with Compound 12b (or vehicle) for 3 weeks using 50 mg/kg on a Q2Dx2/week schedule in A). Four days after the last dose (day 21) tumors were harvested and stained for γH2AX (brown staining). B) Data in the graph indicates a statistically significant (shown is mean ±SD; p = 0.0286, two-tailed Mann-Whitney U-test) increase in γH2AX staining following Compound 12b treatment. (TIF) [file pone.0111060.s005.tif]

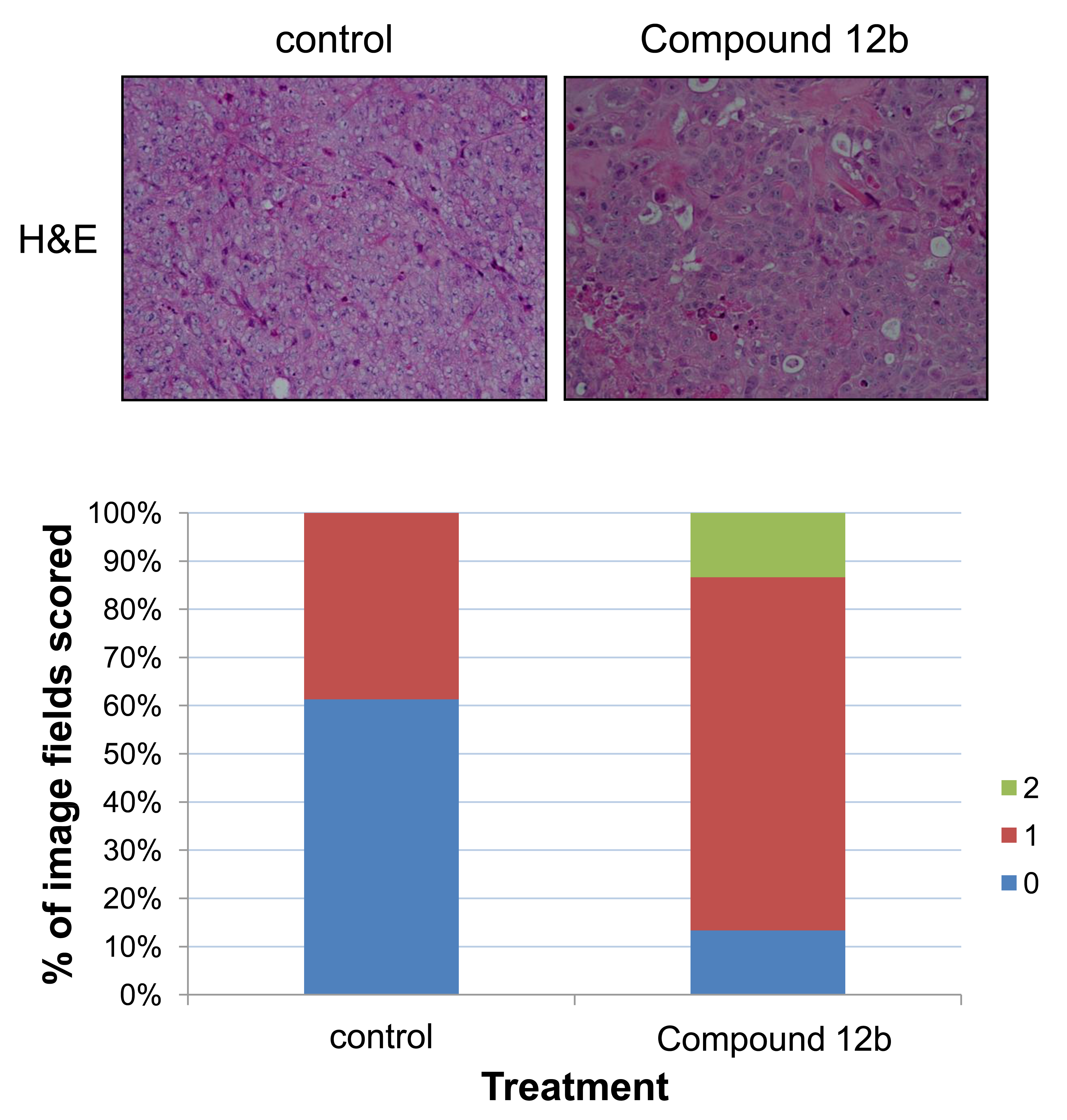

Supplement: Figure S6 — In a separate experiment HCT116 tumor bearing animals were treated orally with 50 mg/kg of Compound 12b on a Q2Dx2/week schedule for 3 weeks. Tumor tissues were harvested at the end of study (day 21) and stained with H&E to assay cell size. Compound 12b treated cells were substantially larger compared to time matched controls. Using a scoring system (see Figure S7), cell size was quantified on a scale from 0–2 with 2 being the largest and 0 being the smallest. Quantification of the data indicate the Compound 12b-treated tumor cells were larger (p = 0.0038, two-tailed Fisher's Exact Test). Values in the graph represent total tumor cell counts from 5 random fields for each tumor, 3 tumors/treatment). (TIF) [file pone.0111060.s006.tif]

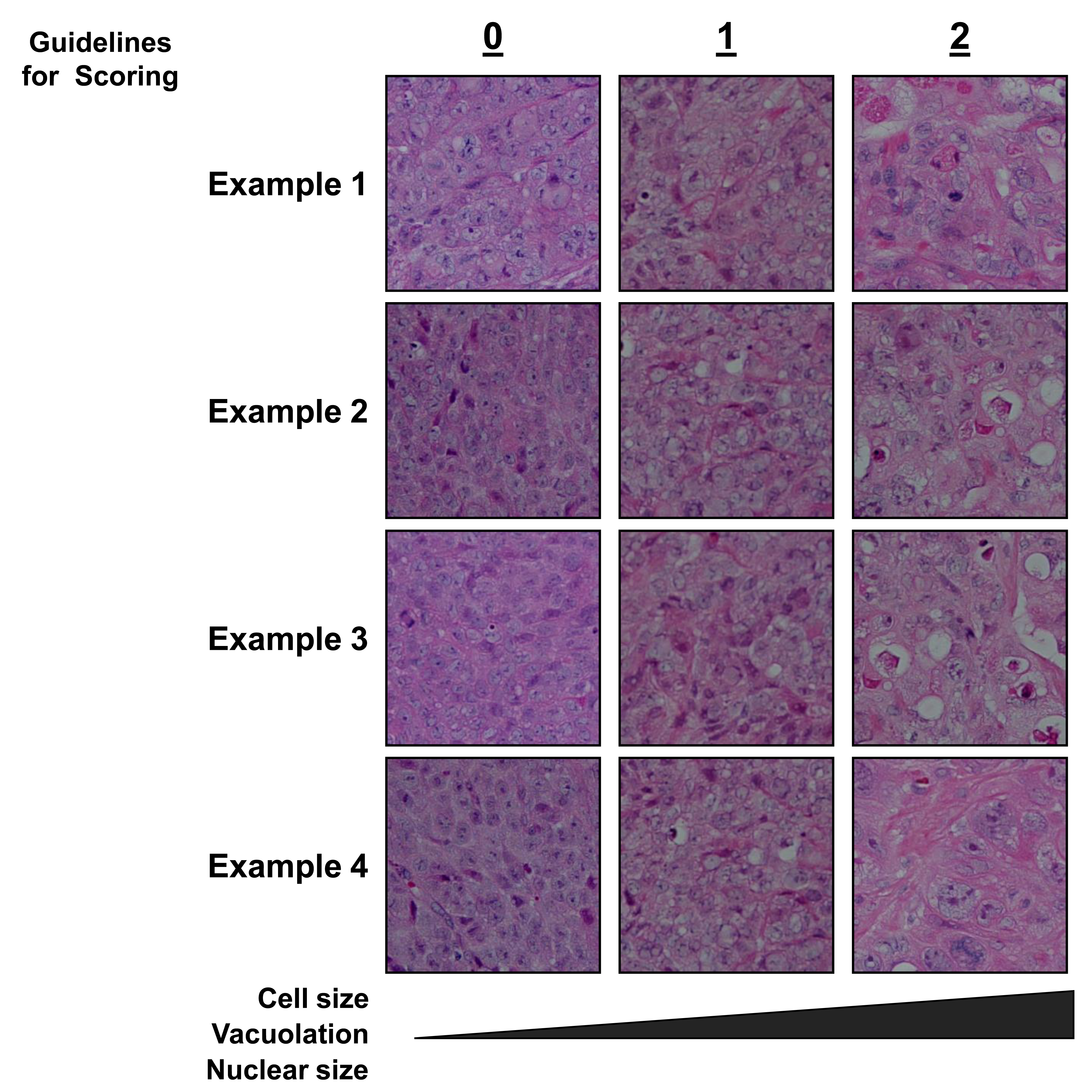

Supplement: Figure S7 — H&E sections showing guidelines for blind scoring of senescence morphology. Four different images were scored in a blinded manner, appearing in random order. Each image was graded on a scale of 0 to 2, with 0 being clearly non-senescent, 1 showing some signs of senescent morphology, and 2 showing clear signs of senescent morphology. The morphological criteria used to establish the scale were: cell size, nuclear size and the degree of vacuolization present in each image. (TIF) [file pone.0111060.s007.tif]

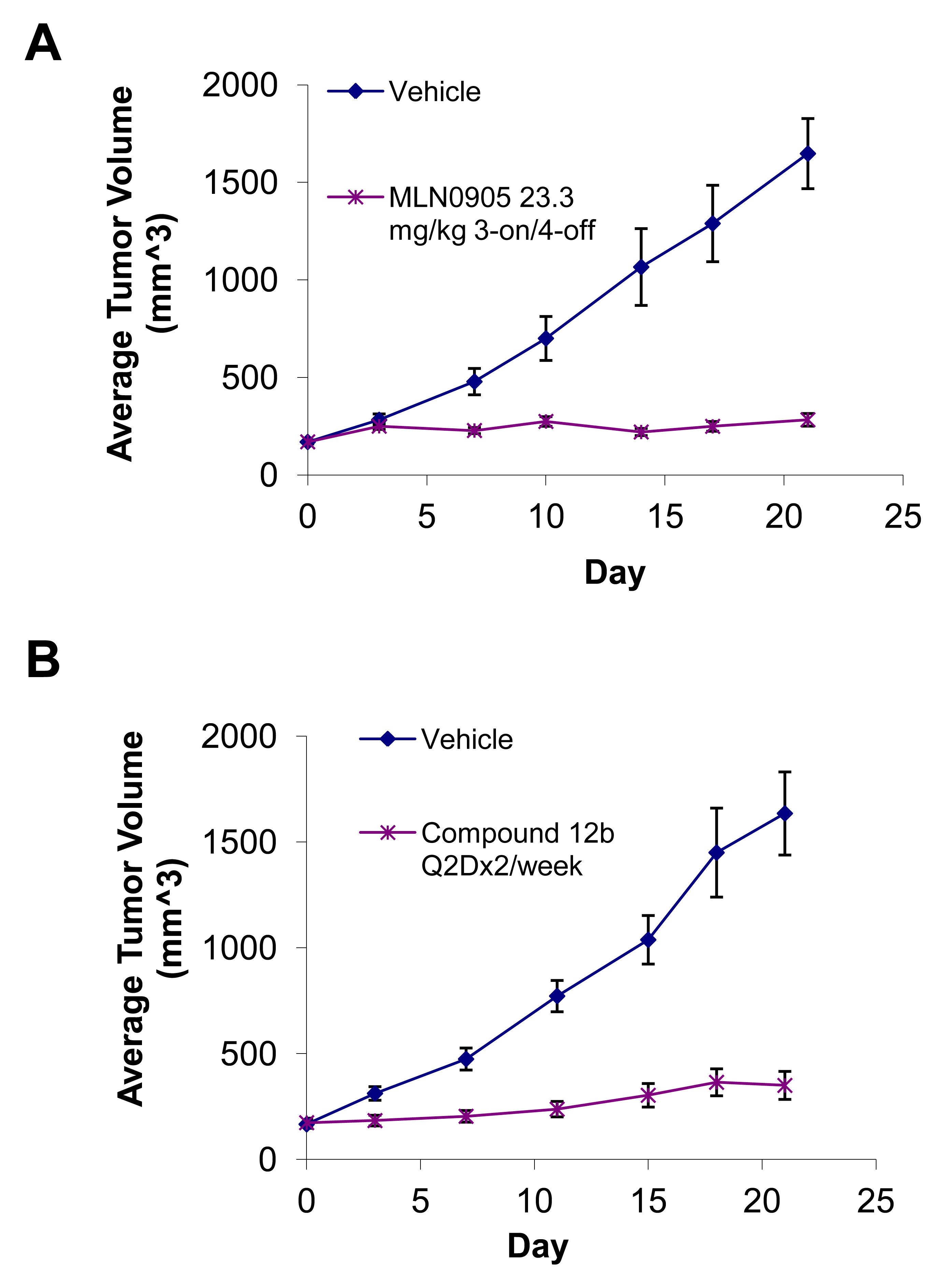

Supplement: Figure S8 — In an efficacy study HCT-116 tumor bearing mice were treated separately with A) MLN0905 and B) Compound 12b using 23.3 mg/kg (QDx3/week) and 50 mg/kg (Q2Dx2/week) for 3 weeks, respectively. Tumor size was measured using vernier calipers and anti-tumor activity was assayed on day 21 of the study. Both MLN0905 and Compound 12b induced significant anti-tumor activity compared to vehicle control (p<0.001). Data shown indicate average tumor volume ±SEM. (TIF) [file pone.0111060.s008.tif]
